# Indications and Early Outcomes for Total Pancreatectomy at a High-Volume Pancreas Center

**DOI:** 10.1155/2010/686702

**Published:** 2010-06-23

**Authors:** Monika S. Janot, Orlin Belyaev, Sabine Kersting, Ansgar M. Chromik, Matthias H. Seelig, Dominique Sülberg, Ulrich Mittelkötter, Waldemar H. Uhl

**Affiliations:** ^1^Department of General and Visceral Surgery, St. Josef Hospital, Ruhr University of Bochum, Gudrunstrasse 56, 44791 Bochum, Germany; ^2^Department of Surgery, Katharinen Hospital Unna, 59423 Unna, Germany

## Abstract

*Background*. This study aimed to analyse the most common current indications for total pancreatectomy (TP) at a high-volume pancreas center. *Method*. Prospectively collected data on indications and short-term outcome of all TP's performed from January 2004 until June 2008 were analysed. *Results*. The total pancreatectomies (TP) were 63, i.e., 6.7% of all pancreatic procedures (*n* = 948). Indications for TP were classified into 4 groups: tumors of advanced stage, *n* = 23 (36.5%), technical problems due to soft pancreatic tissue, *n* = 18 (28.6%), troubles due to perioperative surgical complications, *n* = 15 (23.8%), and therapy-resistant pain due to chronic pancreatitis, *n* = 7 (11.1%). Surgical complications occurred in 23 patients (36.5%). The mortality in elective TP was 6.25%. Median postoperative stay was 21 days. Mortality, morbidity and the other perioperative parameters differed substantially according to the indication for pancreatectomy. *Conclusion.* Total pancreatectomy is definitely indicated for a limited range of elective and emergency situations. Indications can be: size or localisation of pancreatic *tumor, trouble, technical diffuculties and therapy-refractory pain *in chronic pancreatitis. A TP due to perioperative complications (*troubles*) after pancreatic resections is doomed by extremely high morbidity and mortality and should be avoided.

## 1. Introduction

The complete removal of the pancreas has been a topic of controversial discussions ever since surgeons realized that it was feasible. The sporadic reports of total pancreatectomy (TP) in the 1940s and 1950s of the twentieth century grew to a frank enthusiasm about the potential advantages of the procedure with the accumulation of surgical experience in the 1970s but were about to vanish later due to the negative metabolic consequences of the operation.

The complete removal of the gland has already been established as a potential option in the treatment of locally advanced pancreatic cancer, multifocal or recurrent exocrine, and endocrine tumors [1–4]. Troubles, such as perioperative complications arising from pancreatic anastomotic leakage with or without bleeding or apoplexy of the pancreatic remnant, may require complete removal of the remaining organ in an emergency situation [5, 6]. Extremely soft pancreatic tissue has been described as a technical reason for TP during the primary operation to prevent a high-risk pancreatic anastomosis [7]. Therapy refractory pain associated with chronic pancreatitis has been reported as a further indication for TP [8–13].

Despite increasing demand for TP and increasing rate of its performance at large pancreas centres recently, reports in the literature on its current indications and results remain scarce [14, 15].

The aim of this study was to analyse the current indications and the outcome between the different indications for TP at a single high-volume institution and determine the current place of TP in the spectrum of pancreatic resections.

## 2. Patients and Methods

All cases of TP were analysed within the prospectively managed pancreas data bank at the Department of General and Visceral Surgery, St. Josef Hospital Bochum, Germany. All resections were performed by three experienced pancreatic surgeons. The set of data available for every patient included detailed information on all preoperative diagnostic procedures, perioperative parameters, and postoperative complications, as well as strict follow-up documentation. The preoperative risk assessment was graded according to the American Society of Anaesthesiologists classification (ASA). Operation time, perioperative blood loss, necessity of red blood cell transfusions, and postoperative hospital stay were evaluated. Postoperative complications were determined and categorised into major and minor surgical complications and nonsurgical ones. Major complications comprised biliary leakage, postoperative bleeding, intraabdominal abscess, and burst abdomen. Minor surgical complications included delayed gastric emptying, cholangitis, chylous fistula, and wound infection. Nonsurgical complications were defined as complications within 30 days of surgery or during the hospital stay that were not directly related to the surgical procedure, for example, pneumonia, urinary tract infection, or systemic infections not related to the surgical procedure (e.g., central venous infection). Any death during the hospital stay or within the first 30 days after operation was defined as postoperative mortality.

### 2.1. Statistics

Statistical analysis was performed using the SPSS 16 software package (SPSS Inc., Chicago, IL, USA). All data are presented as median with interquartile range and minimal and maximal values, which are shown in the figures as box-and-whisker plots, respectively. For comparison of quantitative variables were used the nonparametric Mann-Whitney and Kruskal-Wallis tests. Two-sided *P* values were always computed, and an effect was considered statistically significant at *P* < .05.

## 3. Results

### 3.1. Study Population

During the study period of 54 months (January 2004–June 2008) 948 patients underwent surgery for pancreatic disease, of which 599 (63.2%) pancreatic resectional procedures. The total pancreatectomies were 63, that is, 6.7% of all pancreatic procedures. They were performed in 34 (54%) males and 29 (46%) females at a median age of 69 (38–87) years. In 45 cases (71.4%) a TP was performed as a primary procedure; in the rest 18 patients it was a completion pancreatectomy. A splenectomy was performed in 45 patients; in eighteen the spleen was preserved. Twenty-five patients (39.7%) were classified as ASA I -II, while 38 (60.3%) were categorized as ASA class III-IV. Substantial cardiac comorbidity was present in 13 cases (21%), pulmonary in 8 (12.7%), and renal in 4 (6.3%). Insulin-dependent diabetes mellitus was present in 11 patients (17.5%).

### 3.2. Indications for Total Pancreatectomy

The indications for a TP were grouped to the *“Four T's”* categories as summarized in Table 2. 

#### 3.2.1. Tumors

Twenty-three patients (36.5%) underwent TP for malignancy. In 22 cases it was a primary operation and in one patient with a recurrent intraductal papillary-mucinous cancer of the head after left resection a completion pancreatectomy was performed. In 13 cases the main reason for total pancreatectomy was the size of the tumor, which spread over the most of the pancreas—there were 10 T3 tumors and 3 T4 tumors. There was also one patient with a T3 carcinoma of the distal hepatic duct and a positive resection margin on the frozen section—a total pancreatectomy was necessary in that case too. In 8 cases multifocal cancer was found—3 patients had a multicentric intraductal papillary-mucionous carcinoma (all T2), 3 patients suffered a multifocal pancreatic adenocarcinoma (one of them T2 and the other two T3), one patient had simultaneously a cancer of the papilla (T3) and an adenocarcinoma of the pancreatic body (T2), and one patient had a cystadenocarcinoma of the pancreatic head (T3) and a simultaneous undifferentiated neuroendocrine cancer in the pancreatic body. There was only one small T1 cancer of the pancreatic head for which a total pancreatectomy was performed, because of the subtotal atrophy of the rest pancreas due to a severe chronic pancreatitis with preoperatively existing insulin-dependent diabetes mellitus and a severe exocrine insufficiency. There were twelve multivisceral resections (*n* = 12/23, 52%) and four vessel reconstructions in this group (4/23, 17.4%). 3 patients from this group suffered from an endocrine and 2 patients from an exocrine insufficiency of the pancreas preoperatively.

#### 3.2.2. Technical Problems

Eighteen patients (28.6%) underwent total pancreatectomy due to technical reasons, that is, very soft and fatty pancreatic tissue in the remnant. In 13 of those patients the diagnosis was a small cancer of the pancreatic head (six T1 and seven T2), two patients had a T2 cancer of the distal common bile duct, another patient had a benign cystadenoma in processus uncinatus, and there were two cases of intraductal papillary mucinous adenoma in the pancreatic head. There were no multivisceral and no vessel resections in this group. Preoperatively 5 patients had an endocrine and 1 patients also an exocrine dysfunction of the pancreas.

#### 3.2.3. Troubles in the Perioperative Period

Fifteen patients (23.8%) underwent total pancreatectomy because of either early postoperative or intraoperative troubles, that is, complications. Twelve patients underwent a completion pancreatectomy because of postoperative complications after pancreatic resections of the head or tail: in 8 cases that was insufficiency of the pancreatic anastomosis/stump with additional postoperative intraabdominal bleeding in 8 patients; in 2 cases pancreatectomy was necessary because of necrotising pancreatitis of the pancreatic remnant with sepsis, and in 2 patients because of failure of the biliodigestive anastomosis with biliary peritonitis. In three patients the decision to perform a total pancreatectomy was made intraoperatively in an emergency situation due to iatrogenic perforation of the duodenum during ERCP, profuse bleeding of a duodenal carcinoma, and a bleeding of a giant pseudocyst of the pancreatic head with a coagulation disorder. There were *n* = 5/15, 33% multivisceral resections, and *n* = 3/15, 20% portal vein reconstructions in this group. 4 patients had an endocrine and 1 patient an exocrine insufficiency of the pancreas.

#### 3.2.4. Therapy-Refractory Pain

Seven patients (11.1%) suffered from a disabling therapy-resistant pain due to severe chronic pancreatitis with small duct disease. In two patients pancreatectomy was the primary intervention—the first one had a total atrophy of the pancreatic tail, and the second one presented intraoperatively with a large tumor mass, where a malignancy was suspected. In the other five cases a completion pancreatectomy was carried out following prior resective pancreatic surgery without adequate alleviation of pain (two cases after left resection, three cases after pancreaticoduodenectomy). All 7 patients suffered already preoperatively from severe endocrine and exocrine insufficiency and all of them were analgesic drug addicts. Multivisceral resections were necessary in two patients with inflammatory stenosis of the colon. Also there were three cases (43%) with portal vein reconstruction.

A total of 17 (27%) multivisceral resections were carried out. There was one total gastrectomy, ten portal vein resections with direct anastomosis, one resection of the common hepatic artery and reconstruction with the splenic artery, five left or right hemicolectomies, and one left adrenal resection.

### 3.3. Intraoperative Parameters

**Figure 1:**
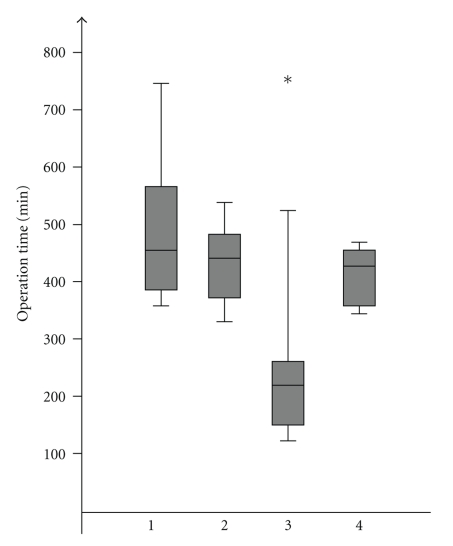
Operation time according to indications: (1) tumor, (2) technical, (3) trouble, (4) therapy-refractory pain, *group 3 significant shorter operation time compared to all others, *P* = .001.

**Figure 2:**
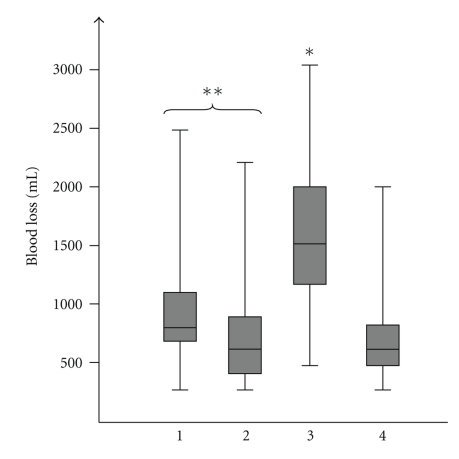
Intraoperative blood loss according to indications: (1) tumor, (2) technical, (3) trouble, (4) therapy-refractory pain, *group 3 significant higher blood loss compared to all others, *P* < .009 in all cases, **group 1 > group 2, *P* = .043.

**Figure 3:**
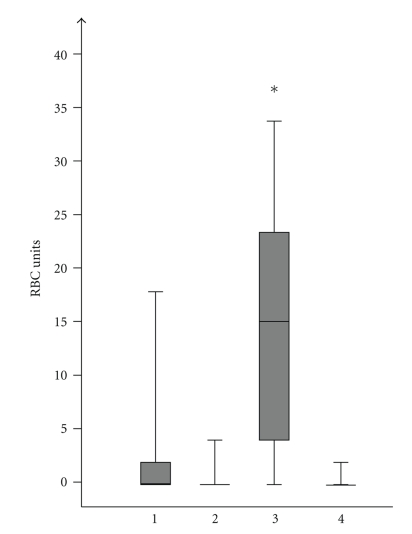
Intraoperative transfused RBC units according to indications: (1) tumor, (2) technical, (3) trouble, (4) therapy-refractory pain, *group 3 significant more transfused RBC units than all other groups, *P* = .001.

Median operation time for the whole group was 420 min; the median intraoperative blood loss was 800 mL. Blood transfusions were necessary in 29 (46%) patients. The duration of the surgical procedure was significantly shorter in patients with troubles as an indication for pancreatectomy with a median time of 210 min compared to all other indications with a median of 448 min (*P* = .001) (Figure 1). At the same time the median intraoperative blood loss in the “troubles” group was with 1500 mL significantly higher than that in patients pancreatectomized because of tumors (800 mL, *P* = .002), soft pancreas (600 mL, *P* = .001), or chronic pancreatitis (600 mL, *P* = .009). Pancreatectomies in the soft pancreas group tended to be performed with less blood loss than those in tumor patients, *P* = .043 (Figure 2). Respectively, transfusion of red blood cell units was also the highest in the “troubles” group with a median of 15 RBC units compared to pancreatectomy due to other indications where the median was zero RBC units (*P* = .001) (Figure 3).

### 3.4. Postoperative Morbidity

Major postoperative complications, either surgical or nonsurgical, occurred in 32 (50.8%) patients. A total of 23 patients (36.5%) developed one or more postoperative surgical complications. The spectrum of minor surgical complications included delayed gastric emptying, cholangitis, wound infection, and chylous fistula. The most often one was delayed gastric emptying—it occurred in nine (14.3%) of the pancreatectomised patients. Cholangitis developed postoperatively three (4.7%) patients. A wound infection was observed in 4 (6.3%) cases and a chylous fistula appeared in 3 (4.8%) patients.

The following major surgical complications were observed: five patients developed an intraabdominal abscess. In three cases the abscess was situated in the upper left abdominal quadrant after splenectomy, another two were found in the left paracolic region. All abscesses were successfully treated via CT-guided drainage. Three patients with emergency completion pancreatectomy suffered an acute postoperative erosion bleeding—in two cases a hemorrhage from the splenic artery, and in the other one from the left gastric artery. Two of these patients needed multiple reoperations. All of those three patients died due to the early or late consequences of the hemorrhagic shock. Three other patients with completion pancreatectomies were reoperated because of failure of the biliodigestive anastomosis. One of those patients developed a malignant postoperative arrhythmia, underwent a pacemaker implantation, but progressed to liver and renal failure with a fatal outcome. Two patients were treated for a wound dehiscence, whereas one of them died due to a progressive multiple organ failure.

The nonsurgical complications comprised of urinary tract infections (most common with 11.1%, 7 patients), 2 cases of pneumonia—one of those patients died due to multiple lung abscesses with respiratory failure and sepsis with multiorgan failure, a pseudomembranous colitis in one patient. One patient died of an acute myocardial infarction. Two patients developed renal failure, which was successfully treated by means of hemofiltration.

### 3.5. Hospital Stay and Mortality

**Figure 4:**
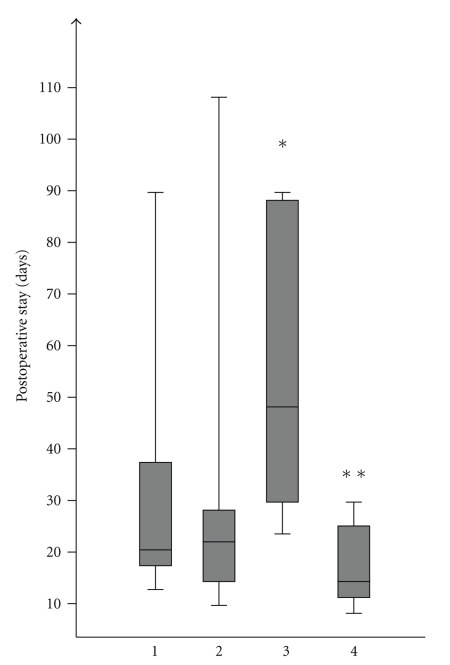
Postoperative stay in 53 patients who survived TP is according to indications: (1) tumor, (2) technical, (3) trouble, (4) therapy-refractory pain, *group 3 significant longer hospital stay compared to 2 (*P* = .04) and group 4 (*P* = .01), **group 4 shorter stay compared to group 1, *P* = .04.

Median hospital stay of all surviving patients (*n* = 53) after TP was 21 days (min–max range 7–108 days). The surviving patients from the “troubles” group had the longest postoperative hospital stay with a median of 48 days, which was significantly longer than the median of all other groups, 20 days, *P* = .035 (Figure 4).

Ten patients (15.9%) died in the postoperative period. Five (7.9%) of them died as a consequence of major surgical complications directly related to the operation, as described above. One patient died of a severe pneumonia with lung abscesses, another one due to myocardial infarction. Another patient died because of thoracic hemorrhage after complicated chest tube insertion for pleural effusion during her stay at the intensive care unit. Two more patients died of multiple organ failure due to MRSA sepsis. Seven of these ten patients were preoperatively classified ASA IV and the other three were ASA III.

The highest mortality (7/15, 47%) was found in the group of patients who underwent TP because of troubles. Three patients died in the “tumor” group, 3/23, 13%. There was no perioperative or late mortality in patients with therapy-refractory chronic pancreatitis (0/7) and in the group with TP for technical or soft tissue-related reasons (0/18). In summery the mortality in elective TP was 6.25% (3/48). A statistically significant difference was found only between the “troubles” and “technical” groups, *P* = .022.

### 3.6. Comparison between Total Pancreatectomy and the Rest Pancreatic Resectional Procedures

A summary of the most important characteristics of the four groups with total pancreatectomy is given in Tables 1 and 2. Table 3 shows a brief comparison of the morbidity and mortality in the TP group versus all other pancreatic resections for the studied period of time. There was no statistical difference with regard to the rate of the most common major and minor postoperative complications. There was a significant difference in the mortality rate. The postoperative hospital stay was statistically longer in cases of TP (*P* = .001). The annual number of TP performed at our center showed a steady increase over the study period, which corresponded to the increase in the total number of pancreatic procedures: *n* = 2/98 in 2004 (2%), *n* = 11/162 in 2005 (6.8%), *n* = 12/220 in 2006 (5.5%), *n* = 20/301 in 2007 (6.6%). In the first six months of 2008 were performed 18 pancreatectomies out of 167 pancreatic procedures (10.8%). The rate of TP was significantly higher in 2008 than in 2004 (10.8% versus 2%, *P* = .008), however the rate of TP (*n* = 47/638, 7.4%) in the second half of the study period (April 2006–June 2008) was not higher than the rate (*n* = 16/310, 5.2%) in the first half (January 2004–March 2006), *P* = .214. 

## 4. Discussion

In 1943/1944 Rocky [16] and Priestly et al. [17] demonstrated the feasibility of pancreatic resection in patients with pancreatic cancer and hyperinsulinism. Ross [7] and Porter [18] were early advocates of TP. They propagated that TP should comprise the standard procedure for pancreatic resection in order to avoid complications of pancreatic anastomotic insufficiency. Furthermore the complete resection of the gland offered an elimination of multicentric disease and the advantage of a wider lymphadenectomy in the case of pancreatic cancer. Using this radical approach many surgeons hoped to improve the overall survival in patients with adenocarcinoma of the pancreas [19]. Several reports demonstrated that TP can be performed safely [14, 15, 20] and with the same morbidity as the standard Whipple resection [8, 21]. In contrast, Trede [22] postulated the complication rate of TP to be threefold higher compared to pancreaticoduodenectomy. Some other studies also showed a significant higher morbidity and mortality in patients who underwent TP in comparison to those who underwent a less extensive resection [2, 23]. The main arguments contra TP were the following metabolic consequences: (1) endocrine insufficiency with a complete insulin deficiency and the necessity of insulin therapy [24], (2) exocrine insufficiency with steatorrhea and the need of durable pancreatic enzyme replacement, (3) the development of steatohepatis with progressive liver failure [25], and (4) the lack of bicarbonate secretion with an increased risk for the development of marginal and peptic ulcers resulting in continuous application of proton pump inhibitors [24].

In 2003 Büechler et al. [14] stated that TP has lost its indication as pancreatic resections could be performed safely with low complication rates. However referring on recent study results Müller et al. [15] partially revised this statemet in 2007. They demonstrated that TP also can be performed safely [12, 26] and underlined the clinical necessity to carry out these operations in some well-selected indications. 

In agree with this recent data of Büechler et al. our study has shown that TP is a demanding surgical procedure. Nevertheless there exist a broad range of indications. We present the data of 63 patients who underwent a TP during the period of 54 months. Indications for TP were analysed and classified in the “*Four T's”: Tumor, Trouble, Technical difficulties and Therapy-refractory pain *in patients with chronic pancreatitis. 

Size or localisation of pancreatic *tumor* can make TP necessary in order to achieve a curative resection. In our series locally advanced or multifocal pancreatic tumors remain the most common indication for TP (40% of our patients). Other examples for tumor-related indications are recurrent pancreatic carcinoma, multicentric cancer [16, 27], intraductal papillary mucinous neoplasia (IPMN) with invasive disease or diffuse involvement [3] of the gland, and extensive neuroendocrine tumors [28, 29]. Although all pancreatectomies in the *“tumor* group” were elective, mortality was relatively high (13%). This may be explained by the high rate of multivisceral resections 52% and vessel reconstructions 17.4% in this group.

In all 15 patients of the *“trouble* group”, TP was either an emergency procedure or an ultima ratio in ICU cases (insufficiency of pancreatic anastomosis, pancreatic stump insufficiency complicated by acute bleeding, necrosis of pancreatic remnant followed by sepsis) in which conservative therapy did not show any success. In those patients who developed vessel arrosion due to insufficiency of pancreatic anastomosis, acute bleeding occurred 5 to 14 days after the initial operation. An early warning signal for imminent vessel arrosion may be a permanent high inflammation parameters with CRP values higher than 100 mg/L. [30–32]. American Society of Anesthesiologists (ASA) classification of patients in the *“trouble* group” was III or IV. In these patients surgery was very demanding due to the poor general condition of these patients, adhesions, and the altered intraoperative situs. The operation time was significantly shorter, but substantially intraoperative blood loss and perioperative need of blood transfusions increased. This group was characterized by the highest morbidity (73%) and mortality (47%). Thus troubles represent disaster and terrible reason for completion and it should be considered alone as an “emergency” choice. Nevertheless only 3 patients died following surgical complications of TP.

The majority of patients died due to medical complications (e.g., pneumonia, myocardial infection, arrhythmia).

In the reported series 18 TPs were performed because of “*technical* difficulties”. In a soft and friable pancreatic remnant that was unable to hold sutures sometimes a safe pancreatic anastomosis was not possible. As 15 % of all pancreatic fistula can lead to a life-threatening haemorrhage and sepsis in these cases, an elective TP was performed. The aim of TP in this group was to avoid an emergency completion pancreatectomy that is associated with a high mortality rate (in our study: 47% cp. *“trouble* group”). There was no perioperative or late mortality in this group. Until today there exists no clear definition of a “soft” pancreas and it remains to be elucidated which tissue characteristics mandate removing the whole gland in the case of technical problems. Definition of objective criteria and the attentive evaluation of pros and cons are necessary to come to the decision to carry out such a “prophylactic” procedure.

Another indication for TP is *“therapy-refractory”* pain. Particularly, in patients with chronic pancreatitis, symptoms not responding to medical treatment can remain or develop again after drainage or resection surgery, indicating progress of the disease or failure of the primary operative procedures, respectively. Several studies demonstrated that only 30–60% of patients with chronic pancreatitis experienced significant pain relief after resection surgery [4, 12]. In these patients TP sometimes is inevitable [8–11]. In our collective 6 patients (13%) underwent a TP because of chronic pancreatitis. In 5 patients a completion pancreatectomy was carried out as reoperation following prior pancreatic surgery as, for example, duodenum-preserving pancreatic head resection (DPPHR). In this group a satisfactory outcome was achieved. Well-reflected and elective planned TP in these patients was performed without any mortality and low morbidity. In this context Müller et al. [15] reported results of 147 TP which correspond well to the findings of our study. 124 patients underwent elective TP and 23 patients completion pancreatectomy for complications. Mortality in the elective group was 4.8%, whereas it was 39.1% in the completion TP group. Operation time of completion TP was significant higher than that in the elective group. Also the number of transfused red blood count (RBC) units was significant higher. Surgical morbidity in the elective group was 24.2%; in the completion TP group it reached 73.9%. Medical morbidity was 14.5% and 56.5%, respectively [15]. These results correspond with the data of our study. Müller et al. [15] reported an increasing demand for TP as 36% of patients underwent TP in the last one of a five-year study period. The number of TP in our collective also increased toward the end of the study period. However this was basically caused by an increased total number of pancreatic procedures that were performed at our hospital. The following trend was observed at our hospital: the number of TP for *tumor* and *therapy-refractory pain* remained constant, the number of *troubles-*TP decreased, and the number of TP by reason of *technical* problems increased.

Several improvements in postoperative management including treatment of diabetes mellitus and substitution of pancreatic enzymes and fat-soluble vitamins have significantly reduced postoperative morbidity and improved quality of life after TP. Recent studies described that there was no significant difference in quality of life in patients with elective TP and partial pancreatectomy [15]. In this context Billings et al. [33] also confirmed no difference in quality of life in 34 TP patients compared with type I diabetic controls. In addition pancreatic surgery has increased its safety, especially at high-volume pancreas centres [15, 33–36], and in future autoislet cell transplantation may offer another option to improve the outcome of patients receiving TP leading to prolonged survival [37, 38].

In conclusion the reported literature and our data exposed a satisfactory outcome for patients when the indication for TP was well reflected. We subdivided the indication of TP into the *“4 T's”: Tumor, Therapy refractory pain, Troubles,* and *Technical* problems. Our data revealed, that TP can be accomplished safely and with complication rates comparable with other resective pancreatic procedures, when it was performed by specialized and experienced surgeons.

## Figures and Tables

**Table 1:** Summary of data related to the four groups of patients who underwent total pancreatectomy (TP).

Group	Number [%]	Operative time [min]	Blood loss [mL]	RBC units	Stay [days]	Mortality [%]
(1) Tumors	23 (36.5%)	450 (360–750)	800 (300–2500)	0 (0–18)	20 (12–89)	3/23 (13%)
(2) Technical reasons	18 (28.6%)	445 (320–535)	600 (300–2200)	0 (0–4)	21 (10–108)	0/18 (0%)
(3) Troubles	15 (23.8%)	210 (120–525)	1500 (500–3000)	15 (0–34)	48 (22–90)	7/15 (47%)
(4) Therapy-refractory pancreatitis	7 (11.1%)	430 (350–480)	600 (300–2000)	0 (0–2)	15 (7–30)	0/7 (0%)

Median (min–max range) for all 63 patients:		420 (120–750)	800 (300–3000)	0 (0–34)	21 (7–108)	10/63 (15.9%)
Median (min–max range) for **elective** TP (48 patients):		440 (320–750)	800 (300–2500)	0 (0–18)	24 (11–109)	3/48 (6,25%)

*For all parameters data are presented as median values with minimum-maximum range.

**Table 2:** Characteristics of 63 patients with total pancreatectomy (TP).

Variable	Tumors	Technical reasons	Troubles	Therapyi-resistant pain
Number	23	18	15	7
Age (median with interquartile range)	66 (62–72)	72.5 (65.5–77.25)	71 (64–75)	50 (46–58)

*Gender*				
Male	10	13	6	5
Female	13	5	9	2

*Histology*				
Cancer	20	15	5	0
Benign tumors	3	3	3	0
Chronic pancreatitis	0	0	6	7
others	0	0	1	0

Primary TP	22	18	3	2
Completion TP	1	0	12	5

Portal vein/SMV resection	9	0	1	2
Splenectomy	20	6	14	5
Pylorus-preserving TP	6	17	9	5
Multivisceral resection	4	0	1	3

Enzyme replacement	2	1	1	7
Endocrine insufficiency	3	5	3	6

*ASA score*				
I	1	0	0	0
II	13	5	2	4
III	8	12	13	2
IV	1	1	0	1

TP indicates total pancreatectomy; SMV: superior mesenteric vein; ASA: American Society of Anesthesiologists.

**Table 3:** Comparison of total pancreatectomy to other pancreatic resections.

Parameter	Total pancreatectomy, *n* = 63	other pancreatic resections, *n* = 536	Statistical difference
Morbidity			
erosion bleeding	3 (4.8%)	16 (3%)	*P* = .447
biliary leakage	3 (4.8%)	8 (1.5%)	*P* = .068
pancreatic leakage	n.a.	25 (4.7%)	n.a.
intraabdominal abscess	5 (7.9%)	26 (4.9%)	*P* = .296
chylous fistula	3 (4.8%)	21 (3.9%)	*P* = .803
delayed gastric emptying	9 (14.3%)	41 (7.7%)	*P* = .072
wound infection	4 (6.3%)	22 (4.2%)	*P* = .409
Mortality in all 63 patients with TP	10 (15.9%)	6 (1.1%)	*P* = .001
Mortality in elective TP (48 patients)	3 (6.25%)	6 (1.1%)	*P* = .006
Postoperative hospital stay [days]	21	14	*P* = .001

*n.a.: not applicable.
